# ICT efficacy and response to different needs in university classrooms: effects on attitudes and active behaviour towards technology

**DOI:** 10.1007/s12528-023-09357-2

**Published:** 2023-01-24

**Authors:** Cecilia Latorre-Cosculluela, Verónica Sierra-Sánchez, Pilar Rivera-Torres, Marta Liesa-Orús

**Affiliations:** 1 Department of Educational Sciencies, University of Zaragoza, 22003 Huesca, Spain; 2 Department of Marketing and Market Research, University of Zaragoza, 50005, Zaragoza, Spain

**Keywords:** Needs, Efficacy, Attitudes, ICT, Structural equation Model

## Abstract

Digital competence is considered to be a crucial learning outcome in education in the 21st century. In this context, research highlights the fact that the perceptions that instructors manifest about different aspects of Information and Communication Technologies (ICTs) condition these professionals’ behaviour towards these learning resources. In the same line, this study aims to analyse the effects that exist between a series of dimensions related to the perception of university teaching staff on the capacity of ICTs to respond to different needs of students, on perceived efficacy and attitudes towards these tools and, lastly, on active behaviour towards their use. To do so, 345 university instructors from the Spanish educational system filled in an online questionnaire. The application of a Structural Equation Model underscores the fact that the ability of ICTs to respond to the different needs of students in the university classroom and their perceived efficacy in the teaching–learning process both exert a positive effect on attitudes in favour of their incorporation into the classroom. In turn, these attitudes also have a significant effect on active behaviour with ICT resources. In addition, various mediating effects are seen to influence an active behaviour. All this gives rise to a discussion on the implications of these results to encourage the training of university teaching staff in the knowledge and management of ICTs. Increasing confidence in ICTs as effective tools to respond to different needs could significantly favour positive attitudes and behaviour so that these resources are actively integrated into the classroom.

## Introduction

For around two decades, the rise in theoretical proposals focused on students, and not so much on the figure of the instructor, has led to a greater mobilization and use of active methodologies within university classrooms. According to what was stated by Silva and Maturana (2016, p.121), active methodologies “materialize this change in the way of understanding learning, since they focus on activities rather than content, which implies profound changes in the actions of instructors and students”. From this point of view, the training process is considered not as an isolated set of tasks and activities that stimulate participation but, rather, as a teaching practice that is made available to students. In this panorama, the unstoppable advance of Information and Communication Technologies (hereinafter, ICT) during the 21st century and the visible explosion in their use that has occurred since the outbreak of the global COVID-19 pandemic both illustrate the inevitable need for technology to permeate the dynamics of institutions of Higher Education (Almarzooq et al., 2020; Van Laar et al., 2017).

After the declaration of the state of alarm by the Spanish Government, all academic activities at all levels of education were immediately suspended in Spain. Following the mandates issued by the competent institutional bodies, an obligatory transition began from face-to-face education to virtual formats that have used ICT as the main communication tools between instructors and students. According to Cáceres (2020), technology has made it possible to create virtual learning environments thanks to which, together with the application of pedagogical and affective methods, instructors have managed to monitor the learning of their students. Among other issues, the exceptional nature of the situation has highlighted the fundamental relevance of the digital skills and competence of instructors, the attitudes of predisposition to their use as part of the teaching–learning process and, in turn, the need to provide didactic support and advice on technology applied to the field of education (Sánchez et al., 2020).

Moreover, another of the biggest challenges that current education faces is the attention to the varied needs of students based on systems that, theoretically, should address the teaching–learning processes with an inclusive vision. In fact, the issue was included as one of UNESCO’s priority sustainable development goals. This was concretized in the need to “guarantee an inclusive and equitable quality education and promote lifelong learning opportunities for all” (UNESCO, 2015, p.6). From a broad perspective and according to Fermín-González (2019), the concept of equal opportunities implies tolerance, quality in teaching, accessibility to learning, respect and social justice. In this regard, the development of inclusive educational processes that advocate authentic attention to the wide ranging needs of students requires a permanent analysis of the processes of change that occur in educational institutions (Fernández-Batanero & Colmenero-Ruiz, 2016). This aspects is understood as the set of all the different needs of students that derive from physical, cultural, social or family conditions. And, in this sense, the response to different needs would be understood as the ability to offer accessibility or providing accommodations for learners.

In certain studies (Turner-Cmuchal & Aitken, 2016; Yu et al., 2016), the role of technology has been highlighted as a vital resource to facilitate learning and as a tool of considerable potential to meet different needs demanded by the versatile society of the 21st century. Likewise, authors such as Yu et al. (2016) have referred to ICT and attention to varied needs as inherent factors in the process of restructuring the educational system. Along the same lines, Freire et al. (2010) have called the ICT–inclusive education dyad as a whole a strategy that offers a large number of opportunities for learning for all students. More specifically, in certain previous studies (Fernández-López et al., 2013) in which technological devices have been used to respond to special needs, it has been found that technology allows adapting to the profile of each student and presenting learning content in a more accessible way. From a more social perspective, also the research by Martin et al. (2021) showed that mobile technology among adults with intellectual disabilities was positively associated with their social inclusion in family contexts, with friends, and at work. Similarly, ICT have also been attributed the potential to promote self-regulated learning and immediate feedback from instructors to their students (Cerrillo et al., 2014). At the same time, when using the infinite possibilities of ICT, the teaching action should consider the individuality of each student and the way in which they learn and process information. Thus, and insofar as they bring with them the possibility of transforming and optimizing learning processes, there is clearly a need to analyse the perceptions and attitudes of Higher Education instructors regarding these ICT tools (Krajka & Kleban, 2014).

Research on the attitudes of instructors towards ICT has a long tradition ever since these tools started to be transferred to the educational domain (Scherer et al., 2018). Thus, as indicated in recent research work (García-Peñalvo et al., 2020), the attitudinal disposition and knowledge of the instructors constitute essential elements of digital competence. In turn, the attitudes of instructors towards ICT presuppose, in line with what was stated by Mirzajani et al. (2016), an outstanding determinant of both the acceptance and the use of these tools in the teaching–learning context.

The analysis of instructors’ attitudes is essential, since it is these agents who must make decisions regarding those measures and strategies that facilitate or hinder their students’ access to different digital resources (Capan, 2012). At the same time, and if the successful integration of ICT into university learning dynamics is expected, the identification and analysis of teaching attitudes is essential (Gilakjani & Leong, 2012). In the context of Higher Education, the accelerated pace at which technology and its multiple applications develop require that the instructor should have enough confidence in its management to allow updated, autonomous and permanent learning (Spante et al., 2018). Thus, and in addition to attitudes towards the use of digital resources, the efficacy perceived by instructors and students of these ICT tools as elements that enhance learning becomes of substantial importance in the teaching–learning processes (Kreijns et al., 2013; Kuo & Belland, 2019).

From the literature published to date (Semerci & Aydin, 2018), a close positive relationship has been evidenced between the perceived efficacy of digital and technological resources and the manifestation of favourable attitudes towards the use of ICT in educational settings. Al-Busaidi & Al-Shihi (2012), and Krumsvik (2011) emphasized the essential need to approach the study of the perceived efficacy of instructors when they are immersed in processes of educational practice with ICT. This author (Krumsvik, 2011) established a differentiation between the trust placed in the use of ICT in an independent way and the trust in the use of ICT for didactic or teaching purposes. According to Siddiq et al. (2016), it might seem as if the active behaviours of instructors towards ICT depend on their perceptions of the capacity or efficacy of these tools to enhance student learning. Similarly, and as highlighted in the work by Teo (2014), the intention to use ICT could be conditioned by the perceived efficacy of these digital resources.

In short, the perceived efficacy maintained a positive relationship with the increase in the levels of commitment in teaching. These perceptions, and an example of this can be seen in numerous studies (O’Bannon & Thomas, 2014; Vanderlinde et al., 2014; Scherer et al., 2015) can be influenced by many different factors, including sociodemographic variables such as the age and gender of the teaching staff or their years of teaching experience. On the contrary, the findings of other more recent studies (Semerci & Aydin, 2018) do not support said variations based on personal and social factors of instructors.

The considerations of the university teaching staff deserve special attention, as these agents have the enormous responsibility of guaranteeing successful and meaningful learning for their students at a time when online teaching tends to digitize content and rethink learning dynamics. For this reason, this work is of considerable relevance, since it seeks to analyse the relationships between several variables related to the perceived efficacy of ICT for learning, the ability to manage the different needs, attitudes towards their integration into the dynamics of learning and teaching and, lastly, active behaviours regarding the use of ICT as an educational methodology in the university environment. In short, it seeks to establish effects between elements that condition and favour the use and exploitation of ICT in the university educational environment in order to know the current situation and promote different initiatives that contribute to the improvement of education and a transformation in which the entire educational community is involved.

## Method

### Sample

The underlying data used in this study were obtained from a sample of N = 345 university instructors in the five macro-areas (Sciences, Health Sciences, Engineering and Architecture, Social and Legal Sciences, and Arts and Humanities) who carry out their teaching in different faculties (Table 1) (age: M = 48.75 years, SD = 10.17 years). The ages of the teaching staff were divided into three intervals: 36.1% were aged between 24 and 45 years, 33.7% were between 46 and 54 years old and 30.2% were between 55 and 73 years of age. Approximately half of the participants were instructors with some kind of permanent connection with the university system (49%) (Full professor and Senior lecturer). The other half is made up of non-tenured teaching staff (23.5%) (Assistant lecturer and Graduate Teaching Assistant) and adjunct teaching staff (27.5%). Three intervals were also established regarding the years of teaching experience in the field of Higher Education: 33.7% of the teaching staff with 0–10 years of experience, 37.8% with 11–25 years, and 28.4% with 26–49 years of teaching experience. Finally, it is observed that 71.3% of instructors teach in the first three years of university degrees, compared to 18.6% in the fourth year and 10.1% at the master’s degree and doctorate levels. The composition of the sample resulted from a kind of accidental non-probabilistic sampling (Tójar & Matas, 2009). Consequently, the sample is made up of those instructors who voluntarily agreed to participate in the research.

**Table 1:** Sociodemographic characteristics in the sample (N = 345)

Variable	n	% of the sample
**Professional profile**		
* Tenured Teaching Staff*	169	49
* Non-tenured Teaching Staff*	81	23.5
* Adjunct Lecturer*	95	27.5
**Knowledge area**		
* Arts and Humanities (A&H)*	53	15.4
* Sciences (S)*	57	16.5
* Health Sciences (HS)*	55	15.9
* Social and Legal Sciences (C&LS)*	121	35.1
* Engineering and Architecture (E&A)*	59	17.1
**Years of teaching experience** (M = 17.96, SD = 11.79)		
* 0–10 years*	115	33.7
* 11–25 years*	129	37.8
* 26–49 years*	97	28.4
**Age** (M = 48.75, SD = 10.17)		
* 24–45 years*	122	36.1
* 46–54 years*	114	33.7
* 55–73 years*	102	30.2
**Year in which more hours are taught**		
* 1st*	90	26.1
* 2nd*	81	23.5
* 3rd*	75	21.7
* 4th*	64	18.6
* Master’s degree / Doctorate*	35	10.1
**TOTAL**	**345**	**100**

### Definition of variables and instrument

After reviewing the literature available to date on the object of this study, all the constructs whose relationships and effects were to be analysed were taken as a starting point. Initially, our first step was to identify and define the measurement indicators of the analysis dimensions from a conceptual point of view. Specifically, some of the sections of the questionnaires from the studies by Agreda et al. (2016); Taquez et al. (2017) were selected. The set of constructs included in the research were measured from perceptual data provided by the teaching staff participating in the research. In order to determine the content validity of the questionnaire, an expert judgement was designed and carried out. A total of five judges, all of whom were professionals working at universities in different disciplines (education, psychology and educational research methods), indicated the adequacy and fit of each indicator, considering the dimension in which it was included.

The perceived efficacy of ICT (EFF) constitutes the first variable of analysis. According to some authors (Friedman & Kass, 2002), this perceived efficacy is defined as the instructors’ perception of their own ability to perform professional tasks (in this case, linked to the use of ICT) and to regulate the teaching process. This variable consisted of a total of eleven indicators related to the perceived efficacy of digital resources such as social networks, web 2.0 applications, cloud storage, device protection software, personal learning contexts, bibliographic managers and online publishing tools.

Second, eleven indicators were established to measure the capacity of ICT to respond to different needs (DIFF) of students. This dimension includes content related to the potential of ICT to promote peer support and collaboration, to consider the strengths and weaknesses of student learning and their interests, and to offer a climate that facilitates the learning process. In turn, other statements are included that refer to the ability of these tools to get students actively involved in problem-solving, to develop their critical thinking, to adjust to different learning rhythms and to stimulate motivation before learning, among others.

Third, attitudes towards ICT (ATT) were defined with a set of seven indicators. Some of the aspects they mention are the perception of these digital resources as elements that enrich the teaching–learning process, the multiple temporal and spatial possibilities they offer for learning, and the fostering of students’ creativity and imagination as well as collaborative work. In turn, perceptions are included regarding the increase in the motivation of both students and instructors, and the improved quality of education, for example. As Cussó-Calabuig et al. (2018) point out, attitudes are defined as those thoughts or feelings that a person can express towards certain issues and that are fundamentally reflected in their way of speaking, acting and behaving towards that particular aspect.

The last variable proposed and analyzed in this study refers to active behavior towards the use of ICT (BEH). This active behavior is defined as the set of actions developed to implement a type of active learning in the classroom and is also affected by the beliefs expressed by people (Siragusa & Dixon, 2008). This dimension was made up of five indicators: creating learning environments with ICT, using digital content to support the teaching–learning processes, using audiovisual systems as material to present the contents, providing ICT tools for planning autonomous learning, and assessing the achievement of subject competences using ICT tools.

For all the dimensions of the study, the items were measured on a Likert-type rating scale with eleven possible responses (0–10). This measurement scale is commonly used by instructors. The figure “0” indicated “totally disagree” with the indicator, while “10” meant “totally agree”. In sum, in this second part of the questionnaire the participants responded to 34 items.

### Investigation and data analysis procedure

The university instructors completed an online survey via the Qualtrics platform, which is widely used for the design and construction of online questionnaires. Participation in the study was completely voluntary and anonymous. After data collection, in a preliminary phase, the researchers generated descriptive statistics of the instructors’ perceived efficacy of ICT tools for improving the teaching–learning process in Higher Education and of the capacity of these digital tools to respond to different needs of students. Likewise, descriptive analyses of instructors’ predisposition attitudes towards the use of digital tools and their proactive behaviours towards the adoption and use of ICT were also included.

The data analysis methodology used in this study is Structural Equation Modelling with latent variables (SEM-LV). This analysis approach makes it possible to include a priori information and to consider its relevance, as well as to reformulate the model that the researcher has initially decided to introduce (Bollen, 1989). All this was carried out taking statistical and robust goodness-of-fit indexes to multivariate non-normality as a reference. The MPLUS software program (Muthén & Muthén, 1998–2015) was used to estimate the models in this study using robust maximum likelihood. Thus, the corrections of Satorra and Bentler (1994) were established both for the goodness-of-fit statistics and for the estimates of the standard errors of the estimated parameters.

In a first phase, the measurement models of the theoretical constructs were tested. To this end, confirmatory factor analyses were performed with latent variables. These dimensions constitute sets of variables that cannot be measured directly and were deduced based on the observed indicators. On the one hand, factorial scores for the perceived efficacy of ICT tools were estimated. The same process was replicated to estimate the factor scores in the dimensions related to the capacity of ICT to respond to different needs, with favourable attitudes towards ICT and with active behaviour towards their educational use. Fornell and Larcker’s (1981) AVE coefficient had a minimum value of 0.50 and McDonald’s (1985) omega coefficient (CRC) yielded a minimum value of 0.70.

After examining this set of models, a structural model was contrasted with latent variables. Different statistics and indices of the overall goodness-of-fit of the model were taken as a reference. Thus, the robust Satorra-Bentler χ² statistic was specified for the proposed model, which is influenced by the size of the sample and the model (Hu & Bentler, 1999). RMSEA, SRMR and CFI were also used. According to Hu & Bentler (1999), an adequate fit of the model would be defined by an RMSEA value between 0.05 and 0.10. The values ​​for the SRMR can vary from 0 to 1, although those models with a more adequate fit obtain values ​​below 0.05. Even with this, a value as high as 0.08 would be within the limits of what is considered acceptable (Hair et al., 2010).

It is proposed that the effects between the variables should satisfy these conditions: first, the perceived efficacy of ICT for improving the teaching–learning process has a direct effect on predisposition attitudes towards the use of technology for educational purposes. Second, the ability of technology to address the varied needs has a positive effect on these same favourable attitudes towards ICT. Third, these attitudes also influence active behaviour towards the integration of ICT as part of educational dynamics. Finally, different indirect effects were also tested within the proposed model. In the set of these effect relationships, the following control variables related to university teaching staff were considered: age, years of experience in university teaching, professional profile and the year in which they teach.

## Results

### Descriptive statistics

Table 2 presents a set of preliminary results in terms of descriptive statistics. Firstly, it is observed that the perception of the capacity of ICT to respond to different needs of university students varies at intermediate levels that exceed the average of 5 (on a scale of 0 to 10). The indicators rated with the highest score refer to the potential that ICT have to allow students to work at their own pace (M = 7.20, SD = 2.35) and to make resources more flexible and thus adjust to the students’ learning (M = 6.98, SD = 2.33). As the least valued, there is the perception that ICT are tools that develop critical thinking in students (M = 5.56, SD = 2.80) and that ICT take into account their strengths and interests (M = 5.69, SD = 2.66). Secondly, and regarding the perceived efficacy of ICT tools to improve the teaching–learning process in Higher Education, it should be noted that the average scores are also around 5, but with some notable differences. First of all, the perceived efficacy of the preparation of materials through multimedia presentations, videos and podcasts (M = 7.54, SD = 2.35) and of the collaborative use of ICT (M = 6.89, SD = 2.69) received the highest ratings. The perceived efficacy of social bookmarking and content syndication (M = 3.20; DT = 2.86) and tools for creating QR codes (M = 3.66, DT = 3.01) are the indicators with the lowest scores. The perceived efficacy of device protection software (M = 5.51, SD = 3.34) and of personal learning environments (M = 5.80, SD = 3.02) were situated at more intermediate levels.

In general terms, the attitudes of university instructors towards the use of ICT are favourable, since average values ​​higher than 6 are detected on a scale of 0 to 10. Specifically, the enrichment of the teaching–learning process with ICT (M = 8.16, SD = 1.95) and the enhancement of collaborative work (M = 8.04, SD = 2.12) received the highest mean scores. Conversely, the lowest scores were obtained in the motivation of ICT for students (M = 6.45, SD = 2.65) and in promoting the implementation of emerging technologies through the use of mobile devices (M = 6.47, SD = 3.06). With regard to the descriptive statistics of active behaviour towards the use of ICT, the means of the indicators of this dimension were found to be positioned with a certain dispersion around the mean of 5. Thus, the university teaching staff shows a strong agreement regarding the use of digital content as support material within the classroom (M = 7.97, SD = 2.26). However, this degree of agreement decreases when they are asked to evaluate the extent to which subject competences are achieved through ICT (M = 4.64, SD = 3.54).

### Measurement model validation

In order to estimate the proposed measurement structures, a confirmatory factor analysis was performed that corresponded to the measurement model. The statistics and goodness-of-fit indices of the measurement models made it possible not to reject these structures (Table 2). Thus, the fit of the confirmatory analyses is reasonable (χ² [523] = 1245.22, RMSEA = 0.07, SRMR = 0.05, CFI = 0.88). Considering the estimates of the parameters, there is evidence of reliability and convergent validity. The set of factor loadings are significant and the coefficients of explained variance (R²) exceed 0.36. Finally, the reliability coefficients of the latent variables exceed the minimum cut-off points, while the minimum value of AVE is 0.49 and that of CRC is 0.70.

**Table 2:** Descriptive statistics and measurement model

	M (Sd)	DIFF	EFF	ATT	BEH	R^*2*^	
**DIFF1**. ICT enable collaboration between classmates.	6.92 (2.45)	0.76				0.58	
**DIFF2**. ICT allow learning from peers.	6.49 (2.64)	0.84				0.71	
**DIFF3**. ICT take into account students’ strengths and interests.	569 (2.66)	0.76				0.58	
**DIFF4**. ICT offer a climate that is conducive to learning.	6.49 (2.58)	0.78				0.61	
**DIFF5**. ICT allow students to make decisions.	6.05 (2.69)	0.81				0.66	
**DIFF6**. ICT allow participation in problem-solving.	6.40 (2.56)	0.79				0.62	
**DIFF7**. ICT allow the development of critical thinking.	5.56 (2.80)	0.78				0.61	
**DIFF8**. ICT can increase the creativity of students.	5.70 (2.84)	0.81				0.66	
**DIFF9**. ICT allow flexible resources with which to adjust learning.	6.98 (2.33)	0.70				0.49	
**DIFF10**. ICT allow students to work at their own pace.	7.20 (2.35)	0.72				0.52	
**DIFF11**. ICT increase student motivation.	6.66 (2.64)	0.75				0.56	
**EFF1**. Social networks.	4.86 (2.98)		0.69			0.48	
**EFF2**. Resources through web 2.0 (blogs, wikis, forums, etc.).	5.98 (2.73)		0.73			0.53	
**EFF3**. Storage within cloud environments (Drive, Dropbox, etc.).	6.77 (2.79)		0.67			0.45	
**EFF4**. Social bookmarking and content syndication to share information.	3.20 (2.86)		0.70			0.49	
**EFF5**. Device protection software.	5.51 (3.34)		0.60			0.36	
**EFF6**. Tools for creating QR codes.	3.66 (3.01)		0.71			0.50	
**EFF7**. Personal Learning Environments.	5.80 (3.02)		0.75			0.56	
**EFF8**. Collaborative use of ICT.	6.89 (2.69)		0.77			0.59	
**EFF9**. Preparation of materials with multimedia presentations, videos, podcasts, etc.	7.54 (2.35)		0.66			0.44	
**EFF10**. Bibliographic managers (Zotero, Mendeley, etc.)	6.05 (3.08)		0.65			0.42	
**EFF11**. Online publishing tools.	4.75 (3.20)		0.80			0.64	
**ATT1**. ICT enrich the teaching–learning process.	8.16 (1.95)			0.77		0.59	
**ATT2**. With ICT, learning happens everywhere and at all times.	7.55 (2.44)			0.70		0.49	
**ATT3**. ICT are motivational tools for students.	6.45 (2.65)			0.82		0.67	
**ATT4**. ICT favour collaborative networking.	8.04 (2.12)			0.71		0.50	
**ATT5**. The use of mobile devices in the classroom encourages the implementation of emerging technologies.	6.47 (3.06)			0.74		0.55	
**ATT6**. The use of ICT increases the motivation of instructors and students.	6.57 (2.67)			0.87		0.70	
**ATT7**. ICT improve the quality of education.	7.11 (2.58)			0.80		0.64	
**BEH1**. I create learning environments with ICT in the classroom.	6.21 (0.76)				0.81	0.66	
**BEH2**. I use digital content as a support within the classroom.	7.97 (2.26)				0.70	0.49	
**BEH3**. I use videos as classroom material to learn.	6.13 (3.48)				0.65	0.42	
**BEH4**. I provide ICT tools for autonomous learning.	5.72 (3.23)				0.70	0.49	
**BEH5**. I evaluate subject competences using ICT tools.	4.64 (3.54)				0.69	0.48	
***α***		0.94	0.91	0.91	0.81		
***CRC***		0.77	0.70	0.77	0.71		
***AVE***		0.59	0.49	0.59	0.50		

χ^2^ [523] = 1245.22; RMSEA = 0.07; CFI = 0.88; SRMR = 0.05

### SEM analysis

After evaluating the dimensional structure of the latent variables, the hypothesized effects in the theoretical model were analysed. The study control variables were also taken into account in this model (Table 3). The goodness-of-fit statistics were sufficiently reasonable to consider that the model fits (χ² [824] = 1809.45, RMSEA = 0.06, SRMR = 0.03, CFI = 0.85). First, it is observed how the perception of the capacity of ICT to respond to different needs has a positive and statistically significant effect on favourable attitudes towards these tools (DIFF: β = 0.84, p < .000). Second, the perceived efficacy of ICT tools within the teaching–learning process also predicts favourable attitudes towards these digital resources (β = 0.21, p < .001). Third, these attitudes exert a positive and significant effect on active behaviour towards the use of technology within the university classroom (β = 0.61, p < .000). Interpreting these relationships between the data, the model would translate into the fact that the more university instructors agree on the capacity of ICT to respond to the wide ranging needs and on their perceived efficacy in the teaching–learning process, the more positive the attitudes towards these resources will be. In parallel, more favourable attitudes will lead to more active behaviour towards the use of ICT.

On considering the direct effects of the control variables of the study on each of the factors included in the model, few significant differences are observed. Regarding the professional role, compared to that of tenured teaching staff, the perceived efficacy of ICT by instructors with a non-tenured or adjunct contract is significantly higher (non-tenured teaching staff: 0.17, p < .05; adjunct lecturers: 0.34, p < .000). In the dimension of the capacity of ICT to respond to different needs, only instructors with more experience in university teaching (26–49 years) were found to position themselves with a lower degree of agreement compared to the newer instructors (0.23, p < .05). Finally, instructors who teach in the fourth-year report significantly more positive attitudes towards ICT than instructors in the first year (0.11, p < .05).

**Table 3:** Results of the Structural Model

	DIFF	EFF	ATT	BEH
*DIRECT EFFECTS*				
Professional profile				
* Non-tenured teaching staff*	0.09	0.17*	− 0.01	0.01
* Adjunct lecturer*	0.16	0.34***	0.03	0.02
**Year**				
* 2nd*	− 0.02	− 0.14*	0.06	− 0.02
* 3rd*	− 0.01	− 0.01	0.04	− 0.01
* 4th*	− 0.03	0.00	0.11*	− 0.11
* Master’s degree / Doctorate*	− 0.04	0.01	0.03	0.02
**Age**				
* 46–54 years*	− 0.04	− 0.02	− 0.01	− 0.07
* 55–73 years*	− 0.05	− 0.00	0.03	− 0.07
**Years teaching experience**				
* 11–25 years*	− 0.04	0.02	0.05	0.09
* 26–49 years*	− 0.23*	− 0.06	0.05	0.05
*** DIFF***			0.84***	
*** EFF***			0.21**	
*** ATT***				0.61***
***INDIRECT EFFECTS***				
*** DIFF***				0.52***
*** EFF***				0.13**
***R***^***2***^	0.13	0.16	0.78	0.13
***Goodness-of-fit***:	χ^2^ [824] = 1809.45; RMSEA = 0.06; CFI = 0.85; SRMR = 0.03			

### Mediation analysis

**Fig. 1:**
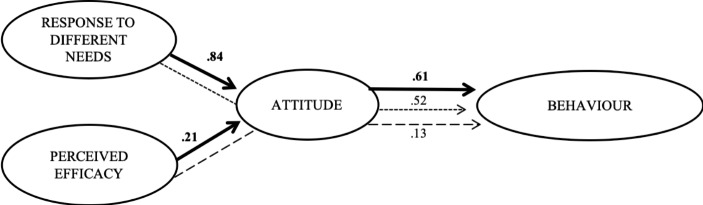
Diagram of results and effects of the Structural Model

In addition to the direct effects, both the total and specific indirect effects of the predictor variables on the outcome variables were also tested. Firstly, the results reveal a significant mediating effect of the positive attitude towards ICT on the relationship established between the variables of the perceived ability of ICT to respond to the varied needs of students and active behaviour towards the use of technology (β = 0.52, p < .000). This fact would be interpreted as indicating that the effects of the capacity of ICT to respond to different needs on active behaviour could be increased if they were translated into an improvement in attitudes towards ICT. Secondly, attitudes also significantly mediate the relationship between the perceived efficacy of ICT in the teaching–learning process and active behaviour towards its use in the university classroom (β = 0.13, p < .001). Consequently, the interpretation of these relationships alludes to the fact that, in order to increase the effect established between these two variables (perceived efficacy and behaviour), the need to improve attitudes towards ICT should be stressed. When the mediating variable (attitudes towards technology) is included in the model, the direct effects of the capacity to respond to varied needs of students and efficacy on behaviour are less than the total effect. Consequently, a partial mediation model would be considered. Figure 1 shows a visual synthesis of the direct and indirect effects of the model under test.

## Discussion

The purpose of this research was to the look for relationships between the capacity of ICT to respond to different needs of students, the efficacy perceived by instructors regarding their use and their contribution to learning, as well as the active attitudes and behaviours of university instructors to integrate ICT into the teaching–learning process. Likewise, the effects, both direct and indirect, between the different variables under study were also analysed. The results obtained, in general terms, have shown the existence of positive and significant effects among the variables analysed.

One of the results obtained in this study that stands out is the perception of the capacity of ICT to respond to the wide ranging needs of students, as well as the perceived efficacy of ICT by university instructors as conditioning factors that promote a more positive attitude towards their use in the educational process. In this regard, the results of this study reveal the high degree of agreement among university instructors regarding the ability of ICT to adapt to the pace at which students work and learn, regardless of their individual characteristics. Several authors (Castañeda et al., 2015; Fermín-González, 2019; Fernández-Batanero & Colmenero-Ruíz, 2016) have recognized this potential of ICT to attend to the different needs of students. In the same line, in her study related to quality, equity and inclusion in virtual learning Fermín-González (2019) highlighted the importance of designing inclusive educational models that contribute to offering the same learning opportunities to all students through the adequate integration of ICT in the classroom. Similarly, other authors (Turner-Cmuchal & Aitken, 2016; Cáceres, 2020) emphasized the importance of equality and equity in quality education, which in turn contributes to a more inclusive and sustainable education.

The efficacy that instructors perceive from the use of ICT in the classroom has a direct influence on the attitude they adopt towards their use and integration into daily educational practices. This statement has reinforced the conclusions drawn from other research work carried out by different authors (Capan, 2012; Gilakjani & Leong, 2012; Hernández-Ramos et al., 2014; Wu et al., 2017). In fact, the results presented by various authors (Imtiaz & Maarop, 2014; Teo & van Schaik, 2012) have supported the idea that a favourable perception of ICT is positively related to the active use that is made of them in the classrooms at different educational levels. Moreover, in other studies (Zamir & Thomas, 2019) it has been observed that the perceptions, attitudes and motivational disposition towards the adoption of ICT in the classroom produce positive and significant effects on the integration of these tools into the teaching–learning processes. In sum, the results obtained in this study have highlighted the fact that both the perception of the capacity of ICT to respond to different needs of students and their perceived efficacy favour the embracing of positive attitudes towards their adoption and use by instructors. Coinciding with what has been indicated in various studies (Wu et al., 2017), a positive and significant correlation is observed between the attitudinal positioning of instructors towards technological tools and the tendency to use them.

The empirical evidence reviewed has revealed the broad set of authors (Almarzooq et al., 2020; Fernández-Batanero & Colmenero-Ruíz, 2016; Hernández-Ramos et al., 2014; Scherer et al., 2018; Semerci & Aydin, 2018) who have inquired into the benefits of adopting a positive attitude towards the use of ICT in the university classroom. Among the most prominent are the active participation of instructors and the integration of ICT in the teaching–learning process. However, other recent research work (Capan, 2012; Castañeda et al., 2015; Cóndor-Herrera, 2020; Turner-Cmuchal & Aitken, 2016; Fernández-Batanero & Colmenero-Ruíz, 2016; Gilakjani & Leong 2012; Sánchez et al., 2020) has pointed out the importance of the level of digital competence of teaching staff as an essential factor to favour a positive attitude towards ICT, as well as contributing to their use and proper integration in educational practices. Furthermore, Fraillon et al. (2014) stressed that the digital training of instructors should favour the use of ICT in the classroom for pedagogical purposes that contribute to improving the teaching–learning process. Likewise, other studies (Hammond et al., 2011; Gilakjani & Leong, 2012; Cáceres, 2020; Sánchez et al., 2020; Zamir & Thomas, 2019) emphasized the need to provide instructors with guidance and assistance throughout the process of integrating ICT into the classroom. The intention underlying this help would be to accompany them and ensure successful results that encourage their motivation towards these resources and take advantage of the large number of opportunities that they offer in the educational context. From a broader perspective, therefore, it becomes clear that it is important to offer a solid digital training that provides instructors with the strategies they need to transfer all the benefits and opportunities that ICT offer to their students. The vision that instructors have about the usefulness of ICT will also favour a positive predisposition towards their integration into their educational practices for pedagogical purposes.

## Conclusion

In recent decades, the appearance of ICT in our society has brought about its transformation at all levels, and especially so in education. However, the latest events that have occurred since the start of the COVID-19 pandemia have imminently accelerated the integration of ICT into the educational process as a way to face the challenge of the virtual mode of education. At the present moment, the urgent need to integrate ICT into the educational field has become evident, and more so than ever. In fact, the possible advantages offered by the online mode of education over the traditional one have been assessed and, as a result, the challenges that the educational community must face in order to progress towards online teaching have also come to light. Among the most prominent are the digital competence of instructors, access to technological resources, as well as the instructors’ attitude towards the integration of ICT into their pedagogical practices through what are known as active methodologies (Zamir & Thomas, 2019; Wu et al., 2017; Silva & Maturana, 2016; Cóndor-Herrera, 2020). This has evidence the insufficient training in the area of computer science to which most of the instructors in the Spanish educational system have been exposed. For this reason, it is recommended that this casuistry should be taken as a turning point to address those more neglected aspects that need to be reinforced in order to sketch out an educational system capable of adapting to the demands of the new society. As has been reflected in the most recent studies (Wu et al., 2017; Turner-Cmuchal & Aitken, 2016; Sánchez et al., 2020), the educational context is directed towards a type of online teaching in which the integration of ICT into the educational model is an essential requirement to transform learning processes and to achieve success under the new paradigm of quality education, equity, equality and sustainability.

The results from this study have clearly shown that instructors perceive ICT as tools that favour the adaptation of learning to the individuality of students. In the same way, the efficacy that they perceive about their use within educational practices has also been revealed. Both have been proclaimed as indicators of a positive attitude of the instructor towards the integration of ICT into the classroom. In the same way, it has been observed that this positive attitude on the part of the instructor can contribute to a more active behaviour in its use in the classroom by promoting the application of active and innovative methodologies that allow a coherent response to the needs demanded by 21st century society.

This approach requires, among other issues, a consistent methodological transformation that involves all the agents responsible for the educational process in an attempt to adapt to the changes that occur in the context in which they work. With all this, the orientation taken by the university should continue in the line of offering training in how to adapt to new changes and situations, in learning to live and coexist harmoniously, and in the satisfactory incorporation into the labour market. Bryson & Hand (2007) reaffirmed this idea by pointing out that university students engage in a more active way when they have the support of teaching staff who design attractive and stimulating learning environments with the support of ICT that require high achievement results and prompt them to challenge their own thoughts.

Given the representativeness of the sample of participants, the results found here could be generalized to the population of university professors in the Spanish context. Regarding the limitations of this research, although a confirmatory factor analysis has been carried out with the data obtained, in no case could the existence of a cause-effect relationship between the study variables be firmly affirmed. In addition, future research should be developed to understand in a deeper way the possible causes that lead to the manifestation of certain attitudes and behaviours towards technology. All this must be done to improve the praxis and the ability to teach through the use of technology of these professionals. Future research could also include other variables linked to the professional work carried out by instructors, such as the type of relationship with their students or the collaboration with the rest of the teaching staff in higher education institutions.

## References

[CR1] Al-Busaidi K, Al-Shihi H (2012). Key factors to instructors’ satisfaction of learning management systems in blended learning. Journal of Computing in Higher Education.

[CR2] Almarzooq, Z., Lopes, M., & Kochar, A. (2020). Virtual learning during the COVID-19 pandemic: a disruptive technology in graduate medical education.Journal of the American College Cardiology. 10.1016/j.jacc.2020.04.015 PMC7159871 32304797

[CR3] Agreda, M., Hinojo, M. A., & Sola, J. M. (2016). Diseño y validación de un instrumento para evaluar la competencia digital de los docentes en la Educación Superior española. *Píxel-Bit. Revista de Medios y Educación, 49*, 39–56. Retrieved from https://recyt.fecyt.es/index.php/pixel/article/view/61713

[CR5] Bollen KA (1989). Structural equations with latent variables.

[CR6] Bryson C, Hand L (2007). The role of engagement in inspiring teaching and learning. Innovations in Education and Teaching International.

[CR7] Cáceres, K. F. (2020). Educación virtual: creando espacios afectivos de convivencia y aprendizaje en tiempo de COVID-19. *CienciAmérica, 9*(2).

[CR8] Capan, S. A. (2012). Teacher attitudes towards computer use in EFL classrooms. *Frontiers of Language and Teaching*, *3*, 248–254. Retrieved from https://www.academia.edu/2442327/Teacher_Attitudes_towards_Computer_Use_in_EFL_Classrooms

[CR9] Castañeda L, Román MM, Barlam A (2015). Mundos virtuales e inclusión social y educativa: Estudio de caso en el IES Cal Gravat. New Approaches in Educational Research.

[CR10] Cerrillo, R., Esteban, R. M., & Paredes, J. (2014). TIC e inclusión en aulas de Educación Secundaria de la Comunidad de Madrid: Análisis de las prácticas docentes en el modelo 1 a 1. *Profesorado. Revista de currículum y formación del profesorado*, *18*(3), 81–97. Retrieved from http://www.redalyc.org/articulo.oa?id=56733846006

[CR11] Cóndor-Herrera, O. (2020). Educar en tiempos de COVID-19. *CienciAmérica, 9*(2).

[CR12] Cussó-Calabuig R, Farran XC, Bosch-Capblanch X (2018). Effects of intensive use of computers in secondary school on gender differences in attitudes towards ICT: a systematic review. Education and Information Technologies.

[CR13] Fermín-González M (2019). Research on virtual education, inclusion, and diversity: a systematic review of scientific publications (2007–2017). International Review of Research in Open and Distributed Learning.

[CR14] Fernández-Batanero J, Colmenero-Ruiz M (2016). ICT and inclusive education: attitudes of the teachers in secondary education. Journal of Technology and Science Education.

[CR15] Fernández-López Á, Rodríguez-Fórtiz MJ, Rodríguez-Almendros ML, Martínez-Segura MJ (2013). Mobile learning technology based on iOS devices to support students with special education needs. Computers & Education.

[CR16] Fornell C, Larcker DF (1981). Evaluating Structural equation models with unobservable variables and measurement error. Journal of Marketing Research.

[CR17] Fraillon, J., Ainley, J., Schulz, W., Friedman, T., & Gebhardt, E. (2014). Preparing for Life in a Digital Age. The IEA International Computer and Information Literacy Study International Report. Cham: Springer. Retrieved from https://research.acer.edu.au/cgi/viewcontent.cgi?article=1009&context=ict_literacy

[CR18] Freire AP, Linhalis F, Bianchini S, Pimentel M (2010). Revealing the whiteboard to blind students: an inclusive approach to provide mediation in synchronous e-learning activities. Computers & Education.

[CR19] Friedman IA, Kass E (2002). Teacher self-efficacy: a classroom-organization conceptualization. Teaching and Teacher Education.

[CR20] García-Peñalvo, F. J., Corell, A., Abella-García, V., & Grande, M. (2020). La evaluación online en la educación superior en tiempos de la COVID-19. *Education in the Knowledge Society, 21*(12), 26–38. Retrieved from https://dialnet.unirioja.es/servlet/articulo?codigo=7403962

[CR21] Gilakjani AP, Leong LM (2012). EFL teachers’ attitudes toward using computer technology in English language teaching. Theory and Practice in Language Studies.

[CR22] Hair, J. F., Black, B., Babin, B. J., & Anderson, R. E. (2010). *Analysis: Global edition, 7th edition* New Jersey: Pearson Education.

[CR23] Hammond M, Reynolds L, Ingram J (2011). How and why do student teachers use ICT?. Journal of Computer Assisted Learning.

[CR24] Hernández-Ramos JP, Martínez-Abad F, García FJ, Herrera ME, Rodríguez-Conde MJ (2014). Teachers’ attitude regarding the use of ICT. A factor reliability and validity study. Computers in Human Behavior.

[CR25] Hu L, Bentler PM (1999). Cutoff criteria for fit indexes in covariance structure anaysis: conventional criteria versus new alterntivas. Structural Equation Modeling: A Multidisciplinary Journal.

[CR26] Imtiaz MA, Maarop N (2014). A review of technology acceptance studies in the field of education. Journal of Technology.

[CR27] Krajka J, Kleban M (2014). E-training in practical teacher development from local to global connections. International Journal of Continuing Engineering Education and Life-Long Learning.

[CR28] Kreijns K, Vermeulen M, Kirschner PA, van Buuren H, van Acker F (2013). Adopting the integrative model of behavior prediction to explain teachers’ willingness to integrate ICT in their pedagogical practices: a perspective for research on teachers’ ICT usage in pedagogical practices. Technology Pedagogy and Education.

[CR29] Krumsvik, R. J. (2011). Digital competence in Norwegian teacher education and schools. Högre Utbilding, 1, 39–51. Retrieved from https://www.researchgate.net/publication/305360830_Digital_competence_in_the_Norwegian_teacher_education_and_school

[CR30] Kuo YC, Belland BR (2019). Exploring the relationship between african american adult learners’ computer, internet, and academic self-efficacy, and attitude variables in technology supported environments. Journal of Computing in Higher Education.

[CR31] Martin AJ, Strnadová I, Loblinzk J, Danker JC, Cumming TM (2021). The role of mobile technology in promoting social inclusion among adults with intellectual disabilities. Journal of Applied Research in Intellectual Disabilities.

[CR32] McDonald RP (1985). Factor analysis and related methods.

[CR33] Mirzajani H, Mahmud R, Fauzi Mohd Ayub A, Wong SL (2016). Teachers’ acceptance of ICT and its integration in the classroom. Quality Assurance in Education.

[CR34] Muthén, L. K., & Muthén, B. O. (1998–2007). *Mplus User’s Guide* (5th ed.). Los Ángeles, CA: Muthén & Muthén.

[CR35] O’Bannon BW, Thomas K (2014). Teacher perceptions of using mobile phones in the classroom: age matters!. Computers Education.

[CR36] Sánchez, M., Martínez, A. M. P., Torres, R., De Agüero, M., Hernández, A. K., Benavides, M. A., Rendón, V. J., & Jaimes, C. A. (2020). Retos educativos durante la pandemia de COVID-19: una encuesta a profesores de la UNAM. *Revista Digital Universitaria, 21*(3). Retrieved from https://www.revista.unam.mx/prensa/retos-educativos-durante-la-pandemia-de-covid-19-una-encuesta-a-profesores-de-la-unam/

[CR37] Satorra A, Bentler PM, von Eye A, Clogg CC (1994). Corrections to test statistics and standard errors in covariance structure analysis. Latent variables analysis: applications for developmental re- search.

[CR38] Scherer R, Siddiq F, Teo T (2015). Becoming more specific: measuring and modeling teachers’ perceived usefulness of ICT in the context of teaching and learning. Computers Education.

[CR39] Scherer R, Tondeur J, Siddiq F, Baran E (2018). The importance of attitudes toward technology for pre-service teachers’ technological, pedagogical, and content knowledge: comparing structural equation modeling approaches. Computers in Human Behavior.

[CR40] Semerci A, Aydin MK (2018). Examining high school teachers’ attitudes towards ICT use in education. International Journal of Progressive Education.

[CR41] Siddiq F, Scherer R, Tondeur J (2016). Teachers’ emphasis on develonping students’ digital information and communication skills (TEDDICS): a new construct in 21st century education. Computers & Education.

[CR42] Silva, J., & Maturana, M. (2016). Una propuesta de modelo para introducir metodologías activas en educación superior. *Innovación Educativa 17*(73), 117–132. Retrieved from http://www.scielo.org.mx/scielo.php?script=sci_arttext&pid=S1665-26732017000100117

[CR43] Siragusa, L., & Dixon, K. (2008). Planned behaviour: Student attitudes towards the use of ICT interactions in higher education. *In Hello! Where are you in the landscape of educational technology? Proceedings ascilite Melbourne 2008*. Retrieved May 25, 2022, from http://www.swaraunib.com/indra/Sistem%20informasi/TPB/siragusa.pdf

[CR44] Spante M, Hashemi SS, Lundin M, Algers A (2018). Digital competence and digital literacy in higher education research: systematic review of concept use. Cogent Education.

[CR56] Taquez, H., Rengifo, D., & Mej?a, D. (2017). *Diseño de un instrumento para evaluar el nivel de uso y apropiación de las TIC en una instituci?n de educación superior* Cali, Colombia: Universidad Icesi.

[CR46] Teo T (2014). Unpacking teachers’ acceptance of technology: tests of measurement invariance and latent mean differences. Computers & Education.

[CR47] Teo T, van Schaik P (2012). Understanding the intention to use technology by preservice teachers: an empirical test of competing theoretical models. International Journal of Humane Computer Interaction.

[CR48] Tójar, J. C., & Matas, A. (2009). Fundamentos metodológicos básicos. En A. Pantoja (Coord.), *Manual básico para la realización de tesinas, tesis y trabajos de investigación* (pp. 129–154). Madrid: EOS.

[CR49] Turner-Cmuchal M, Aitken S (2016). ICT as a tool for supporting inclusive learning opportunities. Perspectivas Internacionales sobre la Educación Inclusiva.

[CR50] UNESCO (2015). Education 2030. Incheon declaration and frame work for action for the implementation of sustainable development goal 4.

[CR51] Van Laar E, van Deursen AJAM, van Dijk JAGM, de Haan J (2017). The relation between 21st-century skills and digital skills: a systematic literature review. Computers in Human Behavior.

[CR52] Vanderlinde R, Aesaert K, van Braak J (2014). Institutionalised ICT use in primary education: a multilevel analysis. Computers Education.

[CR53] Wu YCJ, Pan CI, Yuan CH (2017). Attitudes towards the use of information and communication technology in management education. Behaviour & Information Technology.

[CR54] Yu, B., Ndumu, A., Liu, J., & Fan, Z. (2016). E-inclusion or digital divide: An integrated model of digital inequality. *Proceedings of the Association for Information Science and Technology*, *53*(1), 1–5.

[CR55] Zamir S, Thomas M (2019). Effects of university teachers’ perceptions, attitude and motivation on their readiness for the integration of ICT in classroom teaching. Journal of Education and Educational Development.

